# Amitriptyline–perphenazine therapy for persistent idiopathic facial pain: translational perspectives from a retrospective study

**DOI:** 10.1186/s44158-024-00217-8

**Published:** 2024-12-18

**Authors:** Maurizio Marchesini, Giulia Topi, Cesare Bonezzi, Laura Demartini

**Affiliations:** 1 Department of Anesthesia and Pain Medicine, Mater Olbia Hospital, Olbia, Italy; 2 Department of Clinical-Surgical, Diagnostic and Pediatric Sciences, University of Pavia, Pavia, Italy; 3 Pain Medicine Unit, Department of Mini-Invasive Surgery, IRCCS Maugeri, Pavia, Italy; 4 Anesthesia, Resuscitation, Intensive Care and Pain Therapy, University of Pavia, Pavia, PV 27100 Italy

**Keywords:** Chronic pain, Facial pain, Amitriptyline, Perphenazine drug combination, Receptors, Dopamine D2 antagonist

## Abstract

**Background:**

Persistent idiopathic facial pain (PIFP) can be challenging, both in its diagnosis, which appears to be purely exclusionary, and in its treatment, which currently lacks a gold standard. Amitriptyline is considered a first-line therapy, although not always effective. Recent insights into the role of dopamine in facial pain suggest that a novel therapeutic approach could target the dopamine system.

**Methods:**

This study aimed to retrospectively evaluate the efficacy of treatment with amitriptyline–perphenazine association in patients with severe PIFP. Thirty-one patients were given a regimen dose of amitriptyline–perphenazine at dosages ranging between 10/2 and 20/4 mg and were then retrospectively analyzed. We evaluated the following outcomes, referred to the last week prior to follow-up visits: NRS score for pain intensity (minimum, maximum, and average), the number of attacks, and SF-36 questionnaire for quality of life. Comparisons were made between pre- and post-treatment.

**Results:**

Thirty-one patients over 35 were screened. At baseline, average NRS was 5 ± 0.93 (*CI* 95%: 4.6–5.3), and the median number of breakthrough episodes over last week was 5 ± 1.57 (*CI* 95%: 4–6) with a maximum NRS = 9 ± 0.89 (*CI* 95%: 8–9). After treatment, average NRS was 4.1 ± 0.93 (*CI* 95%: 3.8–4.5; *p* < 0.001), maximum NRS was 6.1 ± 1.60 (*CI* 95%: 5.5–6.6), and the median number of attacks was 4 ± 0.99 (*IC* 95%: 3–4) (*p* < 0.001). Regarding SF-36 questionnaire, the most improved parameters were quality of life related to pain (25.89 ± 12.48 vs 31.19 ± 13.44; *p* < 0.001) and physical function (69.56 ± 17.84 vs 84.17 ± 20.99; *p* < 0.001).

**Conclusion:**

Despite limitations, the pain scores, the frequency of the attacks, and quality of life were found to be significantly improved after treatment. Although results are not broad based given the small sample size, the combination of amitriptyline and perphenazine may be an effective and well-tolerated treatment in patients with PIFP. It is abundantly clear that dopaminergic pathways play a key role in pain modulation, yet the underlying mechanisms have not been fully understood, requiring further investigation.

## Introduction

Atypical facial pain (AFP) was first described by Frazier and Russel [1] in 1924 as a condition distinct from, yet related to, trigeminal neuralgia and migraine, and it remained a recognized term in clinical practice for many years.

The third and latest edition of the International Classification of Headache Disorders (ICHD-3) by the International Headache Society (IHS) provided a new terminology for AFP, i.e., persistent idiopathic facial pain (PIFP). PIFP is described as a “persistent facial and/or oral pain, with varying presentations but recurring daily for more than 2 h/day over more than 3 months, in the absence of clinical neurological deficit.” [2].

PIFP is typically poorly localized, dull, and nagging in quality, and it does not follow the distribution of a peripheral nerve. It usually affects only one side of the face, although up to 40% of cases are reported bilaterally [3].

A key criterion in PIFP diagnosis is that the pain cannot be linked to any other medical condition. Hence, it is considered a diagnosis of exclusion, and as such, it should be differentiated from atypical trigeminal neuralgia, myofascial pain, painful traumatic trigeminal neuropathies, and others [4]. Psychiatric comorbidities are also highly prevalent in PIFP patients [5]. A feature that often puzzles examiners is the inconsistency between the severity of pain reported and the patient’s apparently calm outward appearance.

A typical dental cause, i.e., a cavity or abscess, must also be excluded. A clinical subset of PIFP is “atypical odontalgia” (AO), described as a continuous pain in one or more teeth or in a tooth pocket after extraction without dental causes (i.e., phantom tooth pain) [6]. Often, the pain is preceded by dental treatments; however, these may be unsuccessful attempts to control an orofacial pain that had appeared spontaneously [7].

Due to the generic diagnostic criteria and recent reclassification of chronic facial pain, the epidemiological data are limited and difficult to interpret. According to a 2009 study, the annual incidence of PIFP is 4.4 per 100,000 persons/year [8], and another study estimated its prevalence at 0.03% [9]. PIFP is prevalent in women with a 3:1 ratio, and the average onset age is 45.5 years [10]. An epidemiological study in the UK found chronic orofacial pain to be present in 7% of the population, and these patients displayed frequent comorbidities, such as chronic widespread pain, irritable bowel syndrome, chronic fatigue, high levels of anxiety about their health, and “reassurance-seeking” behaviors [11].

The pathophysiology of PIFP is still obscure. Some authors suggest it may be the result of hyperactivity of central neurons triggered by a peripheral nerve lesion. On the other hand, the significant prevalence of psychological comorbidities and the absence of clearly preexistent nerve damage account for primary chronic pain. In both cases, antidepressants are considered a first-line therapy, as confirmed by a systematic review of literature conducted by the Special Interest Group on Neuropathic Pain (NeuPSIG) of the IASP in 2015. The NeuPSIG gave a strong GRADE recommendation as first-line therapy for TCAs, SNRIs, and/or gabapentinoids in neuropathic pain [12].

Among antidepressants, amitriptyline has shown significant pain relief in patients treated with low doses [13] and has been confirmed as the first-line pharmacological treatment in patients with PIFP [14]. There are a few speculations about the mechanisms by which this effect may be obtained. It might be effective by inhibiting noradrenaline synaptic reuptake in the central nervous system, thereby inducing pain relief [15]. Another possible mechanism is the blocking of voltage-gated sodium channels and consequently the modulation of ectopic firing of neurons [16]. Indeed, amitriptyline is often recommended as an adjuvant treatment in several chronic primary pain states (i.e., fibromyalgia) [17]. New acquisitions in the central mechanisms underlying chronic pain conditions reveal that the dopamine (DA) system may play a role. In the past 20 years, many studies have investigated this topic. Indeed, in patients with fibromyalgia syndrome, the production and release of DA were found to be reduced in the presynaptic neurons in a positron emission tomography study [18]. Similarly, altered DA neurotransmission was associated with pain sensitivity and the affective states in patients with back pain [19].

Overall, chronic pain states may be associated with low dopamine levels in the mesolimbic system. As a matter of fact, dopamine is effective in mitigating pain in low dopamine states such as Parkinson’s disease and restless leg syndrome.

However, the exact role of dopamine and its receptors in the modulation of pain has yet to be described in a precise, unequivocal manner. More than one questions remains as to whether dopamine and D2 receptors play an inhibiting or stimulating role in anti-nociception pathways.

In this perspective, modulating dopamine transmission in central synapsis by blocking D2 receptors, a mechanism usually implied in the treatment of psychosis, may help modulating pain [20]. Here, we retrospectively evaluate the effects of a fixed-dose association of amitriptyline–perphenazine (a D2 antagonist) in the case of PIFP.

Perphenazine is a neuroleptic of the phenothiazine class of the piperazine type. Its mechanism of action is essentially related to the blockage of the D2 dopamine receptor and, to a lesser extent, of the D1 dopamine receptor. This drug also has a strong affinity for serotonin 5-HT2 receptors and histamine, while it has a modest adrenolytic and anticholinergic activity [21].

As for the treatment of chronic pain states, amitriptyline should be started at a dosage of 10 to 25 mg/day and increased by 10 to 25 mg/week to the maximum suggested (75 mg) or tolerated dosage [22]. To minimize the risk of adverse events in the elderly, amitriptyline should be started at a low dosage (10 mg/day) and titrated gradually in 10-mg increments.

Given these premises, the primary objective of the study was to assess the effects of a fixed dose of amitriptyline/perphenazine combination to reduce pain scores and frequency of attacks.

## Methods

### Study design

This is a retrospective observational study of outpatients diagnosed with PIFP who were referred to the Pain Unit of ICS Maugeri in Pavia and were treated with a fixed-dose combination of amitriptyline and perphenazine. Data were retrieved from outpatient electronic medical records. The study was approved by local Ethics Committee of ICS Maugeri, Pavia, Italy, on Feb 4, 2020 (protocol code 2395/2020).

To be enrolled in the study, all patients were thoroughly examined. The overall evaluation encompassed a comprehensive assessment and optimization of conservative management, including neuropsychological testing and appropriate neuroimaging. Both previous and most recent medical history were collected, ruling out the presence of any pathology referring to trigeminal neuralgia, headache/migraine, and systemic diseases that could otherwise explain the persistence of facial pain. All enrolled patients had previously failed first-line therapy with amitriptyline alone, due to lack of efficacy. The minimum dosage to consider treatment with amitriptyline ineffective was 50 mg per day. Any medications other than amitriptyline patients might have been taking were maintained.

The primary outcome of the study was to assess the effects of a fixed dose of amitriptyline/perphenazine combination to reduce pain scores and frequency of attacks; secondary outcomes pertain to the overall well-being, emotional and social implications of chronic pain, and quality-of-life improvements.

All patients were evaluated for their pain intensity as measured by numerical rating scale (NRS) and for their quality of life using a SF-36 questionnaire. We selected patients who underwent a course of amitriptyline (10 mg) and perphenazine (2 mg) in a fixed-dose combination. Usually, the starting dose was one tablet per day; in some cases, dosage had been increased to one tablet b.i.d. after 15 days, if necessary and appropriate.

### Statistical analysis

Since little has been published on the effects of amitriptyline and perphenazine on pain and quality of life in patients with PIFP, a power analysis could not be based on previous research. We performed a descriptive analysis on data relating to pain intensity using a 10-point Likert scale (*NRS*: 0 = no pain, 10 = maximum pain), number of acute episodes, and quality of life (SF-36 questionnaire). Data were collected at baseline (before treatment) and during follow-up visits, at least once a month. A preliminary test of the normality of the data distribution was accomplished with the Kolmogorov–Smirnov test, revealing a non-normal distribution for NRS, whereas a normal distribution was found for the different domains of the SF-36 questionnaire results. The data were presented as mean and standard deviation, when possible. Given the small sample size, both normally and non-normally distributed data were analyzed using the Wilcoxon test for paired data. A *p* < 0.05 was considered statistically significant.

All data analysis and graphs have been performed with R Studio “Spotted Wakerobin” 2022.07.2.

## Results

The data collected refer to patients treated between January and December 2021. Thirty-five patients were preliminarily screened from clinical records by searching for AFP and/or PIFP as primary diagnosis. Patients who fulfilled the previously set inclusion criteria were enrolled consecutively. Indeed, four patients were ultimately excluded because they reached a specific diagnosis other than PIFP (three had primary trigeminal neuralgia with neurovascular compression under radiological investigation; one had atypical cluster headache). Of the 31 remaining patients, 25 were women (80%), and 17 presented AO (54%) (Table 1).

**Table 1:** Demographics

	MED	95% *CI*
Age (years)	51 ± 14.70	46.4–56.7
M/F	6/25	
Pain duration before treatment (months)	14 ± 5.51	12.38–16.26
Length of treatment (months)	4.6 ± 1.89	3.9–5.3

Data are presented as (MED ± SD) and CI (95%)

Two patients stopped the therapy within the first 7 days due to adverse effects (dry mouth and confusion, respectively), while 10 patients (34%) did not require the once-daily regimen to be increased to twice a day. The duration of therapy at the time of follow-up was on average 5 months (minimum 1 month, maximum 7 months). Prior to treatment, all patients had typical course pain, with a baseline mild-to-moderate degree (average NRS = 2.61 ± 0.92), with frequent breakthrough episodes (5 ± 1.57) of very high intensity (mean score NRS = 9 ± 0.89). Complete pre-treatment values are displayed in Table 2.

**Table 2:** Clinical pain intensity scores (minimum, average, and maximum) over the last week measured using a numerical rating scale (NRS) (0–10, “0” indicating no pain and “10” indicating worst imaginable pain) from patients with PFIP and number of attacks pre- and post-pharmacological treatment

	**Pre-treatment**	95% ***CI***	**Post-treatment**	95% ***CI***	***p*** **-value**
NRS min	2.61 ± 0.91	2.2–2.9	1.55 ± 0.85	1.2–1.8	< 0.00001
NRS ave	5 ± 0.93	4.6–5.3	4.16 ± 0.93	3.8–4.5	< 0.00001
NRS max	8.51 ± 0.89	8–9	6.09 ± 1.60	5.5–6.6	< 0.00001
No. of attacks	5 ± 1.57	4–6	4 ± 0.99	3–4	0.00026

Data are presented as mean ± SD and CI (95%). The number of attacks is presented as MED ± SD

**Fig. 1:**
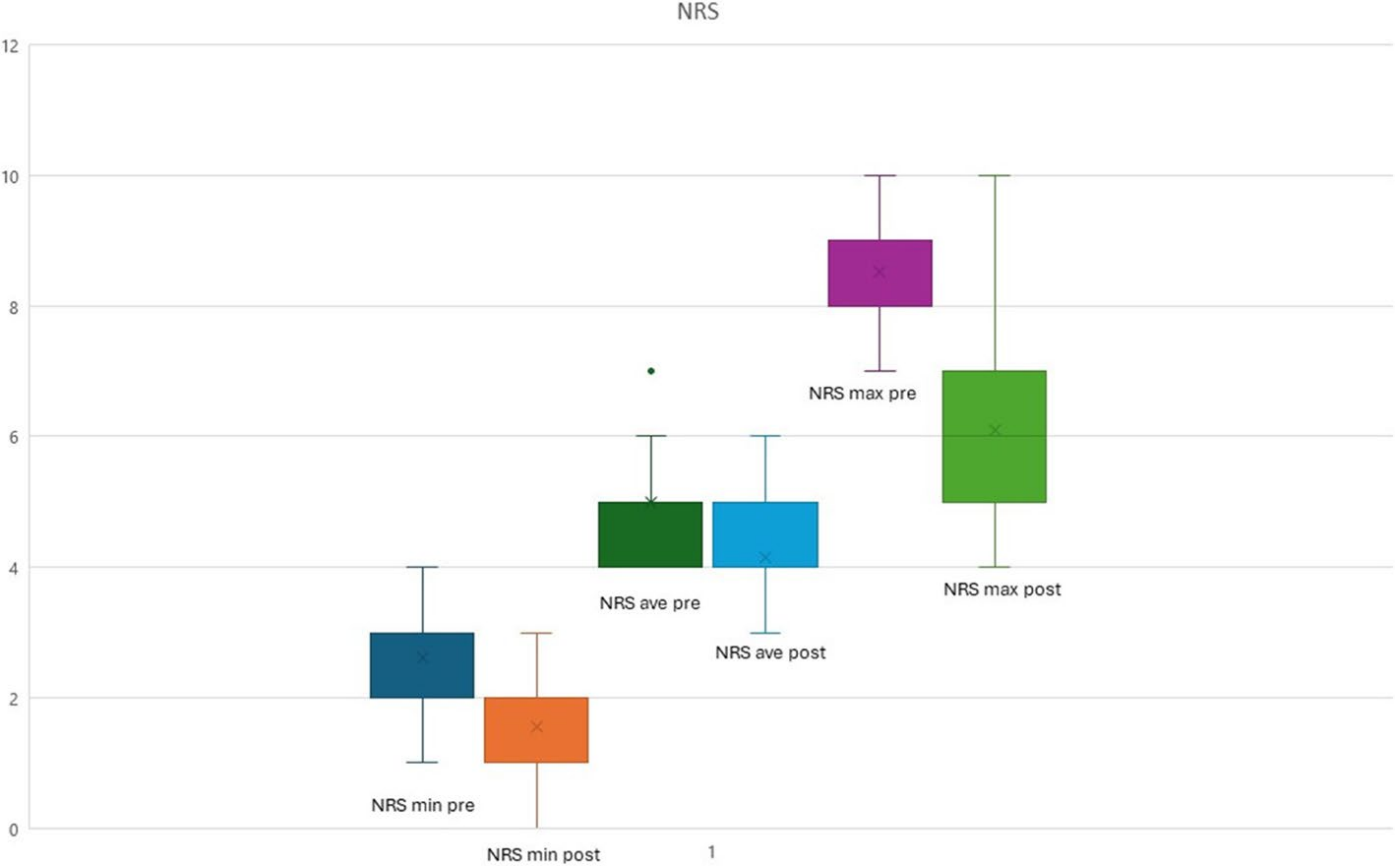
Effects of pharmacological treatment on NRS scores. All the NRS scores significantly decreased after treatment, with a *p*-value < 0.00001 (*NRS* = numerical rating scale 0–10, “0” indicating no pain and “10” indicating worst imaginable pain)

At the time of the baseline visit, all patients had SF-36 values indicative of a significant deterioration in their quality of life. All items were expressions of pathology with evidence pointing to limitations specifically attributable to the emotional sphere (role limitation EP score: 39.34 ± 30.84) and to pain itself (pain score: 25.89 ± 12.48). The items related to the role limitations due to physical and, to a lesser extent, to emotional health highlight the relatively disabling nature of persistent facial pain (see Table 3 for full results). In the follow-up visit, amitriptyline/perphenazine combination therapy showed significant efficacy (Table 2 and Table 3). Indeed, pain measured with the NRS scale showed a statistically significant difference in both maximum, minimum, and mean pain (*p* < 0.00001 for all three measurements) and in the number of attacks (*p* = 0.00026) (Table 2 and Fig. 1). The aspects of quality of life measured by the SF-36 questionnaire were also significantly improved. From the statistical analysis, the least improved aspects, albeit still statistically significant — were the items related to limitations due to emotional reasons and to general emotional well-being. There were no specific questions addressing minor adverse events in case of non-interruption of therapy; during follow-up visits, the main discomfort reported by patients was dry mouth.

**Table 3:** SF-36 pre- and post-pharmacological treatment

	**Pre-treatment**	**CI 95%**	**Post-treatment**	**CI 95%**	***p*** **-value**
Physical functioning	69.56 ± 17.84	60.5–78.6	84.17 ± 20.99	75.8–96.7	< 0.00001
Role limitations PH^a^	21.67 ± 16.42	13.4–30	12.12 ± 10.19	4.8–15.3	0.00009
Role limitation EP^b^	39.34 ± 30.84	23.7–54.9	33.98 ± 26.36	20.5–45.6	0.049
Energy/fatigue	36.37 ± 11.71	30.4–42.3	36.55 ± 14.65	35.1–48.8	0.023
Emotional well-being	42.77 + 11.74	36.8–48.7	42.95 ± 11.83	36.8–48.7	0.048
Social functioning	38.04 ± 17.25	29.3–46.8	53.58 ± 19.14	45.8–63.8	< 0.00001
Pain	25.89 ± 12.48	19.6–32.2	31.19 ± 13.44	24.2–37.2	0.00006
General health	23.30 ± 9.56	8.5–28.1	39.93 ± 9.43	34.7–44.7	< 0.00001

^a^Role limitations due to physical health

^b^Role limitations due to emotional problems. Data are presented as mean ± SD and IC (95%)

## Discussion

To our knowledge, this is the first report of PIFP effectively treated with a therapy that encompasses perphenazine. In our case series, albeit limited, amitriptyline/perphenazine combination was significantly effective to reduce pain intensity and the weekly number of attacks, and it appeared as a well-tolerated drug, with a limited drop-out rate and non-severe side effects. In our cohort of patients, pain significantly interferes with physical functioning, suggesting that coexisting pain states may contribute to physical limitation. Interestingly, the SF-36 items “physical functioning” and “role limitation due to physical health” display posttreatment values in a normal range adjusted for age. By contrast, the items linked to the emotional sphere (role due to emotional problems, energy fatigue, emotional well-being) improved to a lesser extent compared to physical functioning, reflecting in fact the complexity of the perception of pain and its interaction with the psychosocial sphere in humans. To this matter, several recent studies [23, 24] discuss the integration of nonpharmacologic approaches in chronic pain and PIFP management, which proved effective in reducing both the emotional and physical burden of chronic pain, in association with antidepressants.

Although our results are not generalizable given the small sample size, it should be acknowledged that PFIP is a rare condition and other studies have comparable small cohorts [25, 26].

Moreover, our results are demographically coherent with a study defining PFIP especially in regard to clinical characteristics and neuroanatomical findings, PIFP being more prevalent in women (*n* = 25; 80.6%) than in men (*n*= 6; 20%) [27].

These results reinforce that pharmacological manipulation of pain perception is possible, and, indeed, it has neurobiological basis.

### Translational perspectives

Pain, as defined by the International Association for the Study of Pain, is an unpleasant sensory and emotional experience associated with, or resembling that associated with, actual or potential tissue damage [28]. It relies on peripheral signaling pathways and involves several regions of the brain, including the thalamus, the medial prefrontal cortex (mPFC), the striatum, in particular the nucleus accumbens (NAc), the periaqueductal gray (PAG), the insula, somatosensory cortex, and the amygdala.

The important role of neurotransmitters such as norepinephrine, serotonin, and endogenous opioids in pain processing has been widely established, but their revision is beyond the scope of the present work. Here, we want to investigate the lesser-known pathways of dopamine modulation in chronic pain states.

The implication of the dopaminergic system in pain transmission in humans remains controversial, although several studies suggest that dopaminergic pathways can exert either facilitatory or inhibitory pain-modulating effects [29–31].

A conspicuous amount of literature on the matter was reviewed by Changsheng Li and colleagues in 2019 [32]. They concluded that descending dopaminergic pathways play a complex and dualistic role in pain modulation, capable of both inhibiting and facilitating pain, depending on context and CNS location. They suggested that dopamine’s role in pain modulation is significant, particularly within the mesolimbic and mesocortical systems. They also noted that these pathways could be targeted to develop new pain therapies, especially for conditions where conventional treatments are insufficient.

On this matter, dopamine D2 receptor binding in the putamen was in fact found to be associated with pain modulation induced by conditioning stimulation in healthy volunteers [33]. Clinical pathological conditions involving the nigrostriatal dopaminergic system, such as Parkinson’s disease, are often accompanied by pain of central origin [34]. Similarly, altered DA neurotransmission was associated with pain sensitivity and the affective state in patients with back pain [35]. Animal studies also indicate that DA plays a role in central pain modulation [36, 37].

As for facial pain, dysfunctions of the dopaminergic system in the basal ganglia have been associated with chronic orofacial pain conditions in humans. A 2001 PET study demonstrated for the first time in vivo that patients with a chronic orofacial pain syndrome have a dysfunction of the striatal dopamine system [38].

Moreover, diminished levels of DA metabolites have been documented in the cerebrospinal fluid of the trigeminal cistern in facial pain patients [39]. In addition, studies demonstrated diminished [18F] F-DOPA, increased [11C]raclopride uptake, and subsequent decrease in endogenous DA levels in the putamen in burning mouth syndrome patients [40]. Previous neuroanatomical data suggested that both the striatal and the extra-striatal dopaminergic pathways probably participate in the sensory-discriminative and affective dimensions of pain perception as well as in the modulation of nociceptive information [41].

A recent review discussed the analgesic effects mediated by different DA receptors in various regions of the central nervous system, including the spinal cord, striatum, NAc, and PAG [42]. Overall, DA seems to have a general analgesic effect, although a few studies in recent years have suggested that the system might be more complex than previously anticipated, as the successful addition of a dopamine antagonist in a treatment plan for chronic pain, despite the limitations of this study, seems to suggest. The role of DA receptors in pain modulation is not quite linear and is still a matter of debate — an imbalance in DA receptors expression and DA release could be involved in chronic pain, rather than a simple DA depletion.

**Fig. 2:**
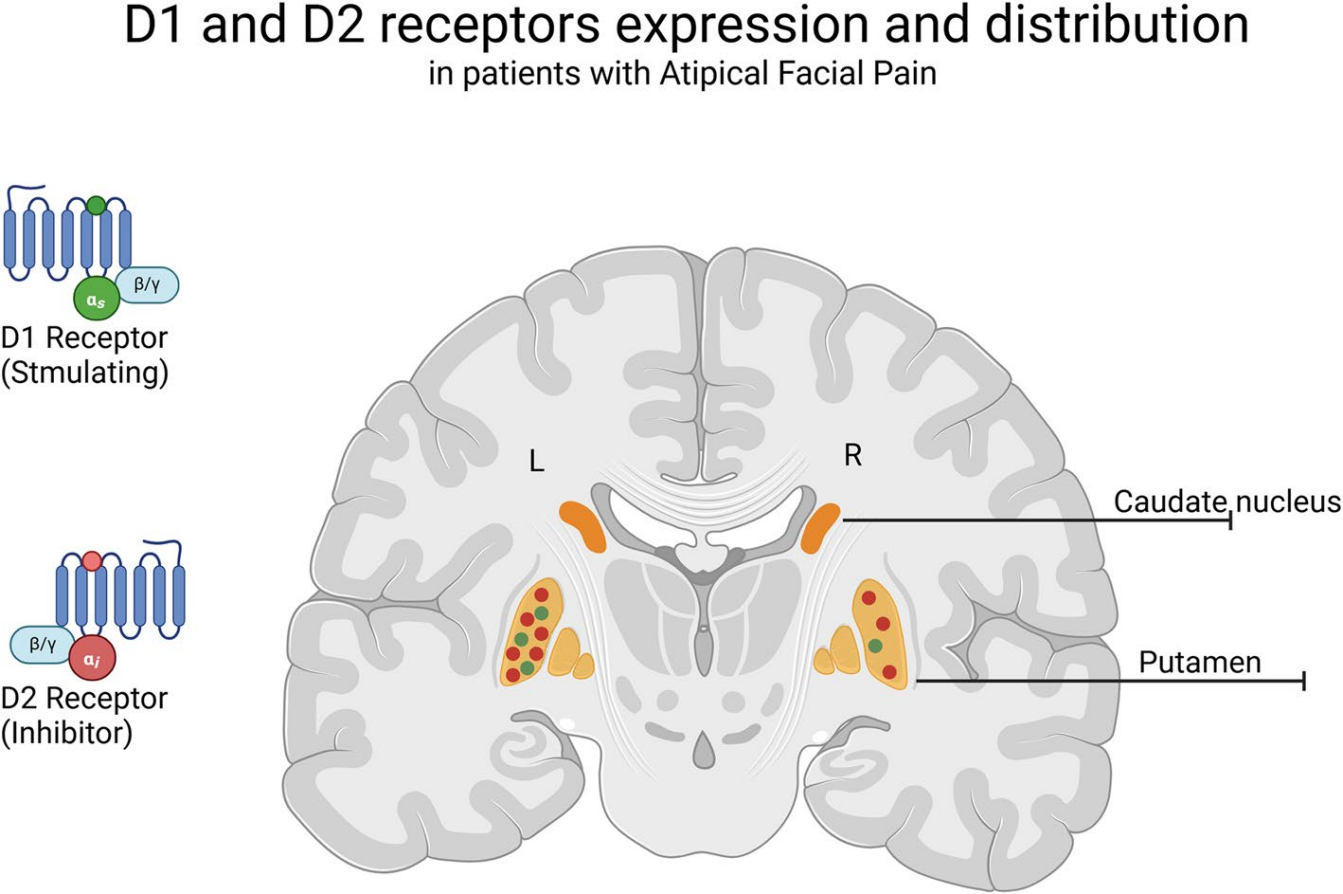
Left putamen and left and right medial thalamus express increased D2 receptors in atypical facial pain patients compared to controls in a PET study. There was also a bilateral decreased D1/D2 ratio in the patient group, suggesting an imbalance in striatal dopaminergic pathways. Created with bioRender.com

In PIFP animal models, PET studies demonstrate the increase in D2 receptor availability in the left putamen and the decrease in D1/D2 ratio in the striatal dopaminergic system [20] (Fig. 2). Similarly, animal studies investigated the role of central DA depletion in neuropathic pain, finding that nigrostriatal DA increased allodynic behavior through D2-like receptors, indicating this as a possible pharmacological target for treating trigeminal allodynia [43].

Notably, eight different dopamine pathways and five different types of DA receptors have been described.

DA receptors have different pharmacological, biochemical, and physiological functions and can be divided into two families: the D1-like family and the D2-like family. When activated, D1-like receptors exert an excitatory activity, while D2-like receptors activation is coupled to the inhibitory Gi protein. Presynaptic D2 receptors also act as auto-receptors to decrease dopamine synthesis and synaptic release. Thus, an increase in D2 receptors availability in chronic pain could explain low levels of DA and at the same time justify the use of D2 antagonists in a multidrug therapeutic approach. In this context, early studies on dopamine metabolism showed that the administration of a variety of D2 receptor antagonists at doses lower than required for antipsychotic effects resulted in an increase in dopamine metabolism [44].

As for dopamine pathways, the role of the nucleus accumbens (NAc) in pain modulation has been recently explored further. The NAc is part of the cortical-mesolimbic pathway and the “reward and motivation” mechanisms, receiving from the ventral tegmental area (VTA). NAc is made up mainly of medium spiny neurons (MSN) containing D1-like or D2-like dopamine receptors [45].

Among the studies that approached the involvement of NAc in pain modulation, Ren and colleagues concentrated on the differences between the NAc core and shell, demonstrating that the NAc core D2-MSNs reduce pain, whereas the NAc shell D2-MSNs exacerbate pain [46, 47]. Dopamine inhibits the D2-MSN in both parts of the NAc; thus, it would influence pain symptoms in a reverse manner: it has a pain-stimulating effect in the core and an analgesic effect in the shell. Notably, the NAc core and NAc shell are innervated by projections from the lateral VTA and the medial VTA, respectively, and project to distinct parts of the ventral pallidum (VP; dorsolateral vs. medial, respectively). All in all, it appears that pain is processed in the basal ganglia by two separate circuits — the pain-relieving lateral VTA–NAc core–dorsolateral VP pathway and the pain-enhancing medial VTA–NAc shell–medial VP pathway — playing contrasting roles.

A 2021 animal model study investigated the roles of dopaminergic and glutamatergic pathways in chronic pain. It demonstrated that dopaminergic signaling via D2 receptors in the dorsal striatum is crucial for dopamine’s analgesic effects, with disruptions in this system diminishing its ability to modulate pain through D2-expressing medium spiny neurons. Additionally, the study highlighted the significance of glutamatergic inputs from the medial prefrontal cortex to the nucleus accumbens (NAc), noting that alterations in this transmission could worsen chronic pain. The findings indicate that distinct dopaminergic circuits connecting the ventral tegmental area (VTA) and NAc regulate pain in opposing ways and interact with different regions of the medial prefrontal cortex, suggesting a complex interplay between dopamine and glutamate in pain modulation [48].

From a clinical perspective, the complexity herein described justifies that diverse clinical chronic states may respond differently to various medications acting on the dopamine system. Therefore, the amitriptyline–perphenazine combination is a promising therapy for patients with PIFP, combining the well-known antinociceptive effects of TCAs with the anti-dopaminergic effects of perphenazine.

In our opinion, these data highlight two points that could be the foundation for further investigations. First, the amitriptyline/perphenazine combination at dosages used in our study was an effective treatment for these patients. Second, the results show a specifically analgesic rather than emotional effect, further corroborating the involvement of the dopaminergic system in pain modulation.

### Study limitations

This study has several limitations:

- The study was retrospectively designed on a limited case series, although the rare occurrence of the disease should be considered. Thus, results are not generalizable.
- The study does not allow for a long-term follow-up of effects of therapy.
- Patients’ follow-up times were not scheduled in advance and vary widely.
- A specific assessment of depressive status before and after the treatment was not conducted.
- Any medications other than amitriptyline that patients were taking were kept unchanged. However, we did not collect data on concomitant limitations.
- We could not collect comorbidities in the baseline characteristics, although this aspect could be relevant as up to 96% of patients with PFIP has psychiatric comorbidities [2].
- NRS and SF-36 may fail to accurately capture the multifactorial dimensions of pain in patients with a high prevalence of comorbidities such as other painful conditions, depressive states, and catastrophism that may affect the overall outcome. A prospective and more complete evaluation is strongly recommended to confirm our preliminary observations.

## Conclusions

Based on the data resulting from our case series, the amitriptyline–perphenazine combination therapy at dosages between 10/2 and 20/4 mg can be considered for PIFP treatment, owing specifically to an analgesic mechanism. The treatment is also well tolerated and not burdened by significant nor frequent side effects. However, a well-designed prospective placebo-controlled study is recommended to confirm these clinical observations.

At present, scientific literature on this topic clearly provides an overwhelming body of evidence indicating that dopaminergic neurotransmission is deficient in chronic pain states. According to the predominant view, this deficiency maintains and exacerbates pain. However, such a general view is not consistent with the findings, both in animal model and in translational research studies, that dopamine promotes pain in some cases. Therefore, it seems that functional changes in the dopamine system during chronic pain and their impact on pain should be considered more in depth, warranting further research.

## Data Availability

No datasets were generated or analysed during the current study.

## References

[1] Frazier CH, Russell EC (1924) Neuralgia of the face, an analysis of 754 cases with relation to pain and other sensory phenomena before and after operation. Arch Neurol Psych 11:557–563

[2] Headache Classification Committee of the International Headache Society (IHS) the International Classification Of Headache Disorders, 3rd edition. Cephalalgia. 2018;38(1):1–211. 10.1177/0333102417738202. 10.1177/0333102417738202 29368949

[3] Kawasaki K et al (2020) Differences in the clinical characteristics of persistent idiopathic facial pain (atypical odontalgia) patients with or without neurovascular compression of the trigeminal nerve. Pain Med 21:814–821 32040150 10.1093/pm/pnz300 PMC7139210

[4] Gerwin R (2020) Chronic facial pain: trigeminal neuralgia, persistent idiopathic facial pain, and myofascial pain syndrome-an evidence-based narrative review and etiological hypothesis. Int J Environ Res Public Health 17:7012 32992770 10.3390/ijerph17197012 PMC7579138

[5] Miura A et al (2018) Psychiatric comorbidities in patients with atypical odontalgia. J Psychosom Res 104:35–40 29275783 10.1016/j.jpsychores.2017.11.001

[6] Woda A, Pionchon P (1999) A unified concept of idiopathic orofacial pain: clinical features. J Orofac Pain 13:172–84; discussion 185-95 10823031

[7] Biçakci S, Öz İ, Sarica A, Giray Y (2005) Neurological and dental aspects of atypical facial pain The Pain Clinic 17:321–325

[8] Koopman JSHA et al (2009) Incidence of facial pain in the general population. Pain 147:122–127 19783099 10.1016/j.pain.2009.08.023

[9] Mueller D et al (2011) Prevalence of trigeminal neuralgia and persistent idiopathic facial pain: a population-based study. Cephalalgia 31:1542–1548 21960648 10.1177/0333102411424619

[10] Ananthan S, Benoliel R (2020) Chronic orofacial pain J Neural Transm (Vienna) 127:575–588 32130516 10.1007/s00702-020-02157-3

[11] Atypical facial pain (2004) a survey of treatment in the Manchester area (UK). Health educational Journal 63:170–188

[12] Finnerup NB et al (2015) Pharmacotherapy for neuropathic pain in adults: a systematic review and meta-analysis. Lancet Neurol 14:162–173 25575710 10.1016/S1474-4422(14)70251-0 PMC4493167

[13] Güler N, Durmus E, Tuncer S (2005) Long-term follow-up of patients with atypical facial pain treated with amitriptyline. N Y State Dent J 71:38–42 16146306

[14] Cornelissen P, van Kleef M, Mekhail N, Day M, van Zundert J (2009) Evidence-based interventional pain medicine according to clinical diagnoses. 3. Persistent idiopathic facial pain. Pain Pract 9(6):443–8 19874535 10.1111/j.1533-2500.2009.00332.x

[15] Su M (2015) Amitriptyline therapy in chronic pain. Int Arch Clin Pharmacol 1:1–5

[16] Wolff M (2016) Amitriptyline and carbamazepine utilize voltage-gated ion channel suppression to impair excitability of sensor dorsal horn neurons in thin tissue slice. An in vitro study Neurosci Res 109:16–27 26945616 10.1016/j.neures.2016.02.006

[17] Alberti FF et al (2022) Comparative efficacy of amitriptyline, duloxetine and pregabalin for treating fibromyalgia in adults: an overview with network meta-analysis. Clin Rheumatol 41:1965–1978 35347488 10.1007/s10067-022-06129-8

[18] Wood PB et al (2007) Reduced presynaptic dopamine activity in fibromyalgia syndrome demonstrated with positron emission tomography: a pilot study. J Pain 8:51–58 17023218 10.1016/j.jpain.2006.05.014

[19] Martikainen IK et al (2015) Chronic back pain is associated with alterations in dopamine neurotransmission in the ventral striatum. J Neurosci 35:9957–9965 26156996 10.1523/JNEUROSCI.4605-14.2015 PMC4495244

[20] Hagelberg N et al (2003) Altered dopamine D2 receptor binding in atypical facial pain. Pain 106:43–48 14581109 10.1016/s0304-3959(03)00275-6

[21] Sweet RA et al (2000) Pharmacologic profile of perphenazine’s metabolites. J Clin Psychopharmacol 20:181–187 10770456 10.1097/00004714-200004000-00010

[22] Overview | Neuropathic pain in adults: pharmacological management in non-specialist settings | Guidance | NICE. http://pathways.nice.org.uk/pathways/neuropathic-pain. 31961628

[23] Eucker SA, Knisely MR, Simon C (2022) Nonopioid treatments for chronic pain—integrating multimodal biopsychosocial approaches to pain management. JAMA Netw Open. 5(6):e2216482 35687341 10.1001/jamanetworkopen.2022.16482

[24] Foerster Z, Kleinmann B, Schlueter N et al (2022) Multimodal pain therapy for persistent idiopathic facial pain - a pilot study. BioPsychoSocial Med 16:25 10.1186/s13030-022-00254-1 PMC9733036 36494736

[25] Sato D et al (2022) Relief of neuropathic pain by cell-specific manipulation of nucleus accumbens dopamine D1- and D2-receptor-expressing neurons. Mol Brain. 15:10 34991655 10.1186/s13041-021-00896-2 PMC8740378

[26] Huang M et al (2022) Dopamine receptor D2, but not D1, mediates the reward circuit from the ventral tegmental area to the central amygdala, which is involved in pain relief. Mol Pain 18:17448069221145096 36464669 10.1177/17448069221145096 PMC9742700

[27] Lazenka MF, Freitas KC, Henck S, Negus SS (2017) Relief of pain-depressed behavior in rats by activation of D1-like dopamine receptors. J Pharmacol Exp Ther 362:14–23 28411257 10.1124/jpet.117.240796 PMC5454591

[28] Raja SN et al (2020) The revised International Association for the Study of Pain definition of pain: concepts, challenges, and compromises. Pain 161:1976–1982 32694387 10.1097/j.pain.0000000000001939 PMC7680716

[29] Wood PB (2008) Role of central dopamine in pain and analgesia. Expert Rev Neurother 8:781–797 18457535 10.1586/14737175.8.5.781

[30] Kim J-YV et al (2015) Spinal dopaminergic projections control the transition to pathological pain plasticity via a D1/D5-mediated mechanism. J Neurosci 35:6307–6317 25904784 10.1523/JNEUROSCI.3481-14.2015 PMC4405552

[31] Liu S et al (2019) Dopamine receptor D2, but not D1, mediates descending dopaminergic pathway-produced analgesic effect in a trigeminal neuropathic pain mouse model. Pain 160:334–344 30325872 10.1097/j.pain.0000000000001414 PMC6344251

[32] Li C, Liu S, Lu X, Tao F (2019) Role of descending dopaminergic pathways in pain modulation. Curr Neuropharmacol 17:1176–1182 31182003 10.2174/1570159X17666190430102531 PMC7057207

[33] Hagelberg N et al (2002) Dopamine D2 receptor binding in the human brain is associated with the response to painful stimulation and pain modulatory capacity. Pain 99:273–279 12237205 10.1016/s0304-3959(02)00121-5

[34] Schott GD (1985) Pain in parkinsonʼs disease. Pain 22:407–411 4047709 10.1016/0304-3959(85)90046-6

[35] Martikainen IK et al (2015) Chronic back pain is associated with alterations in dopamine neurotransmission in the ventral striatum. J Neurosci 35:9957–9965 26156996 10.1523/JNEUROSCI.4605-14.2015 PMC4495244

[36] Megat S et al (2018) A critical role for dopamine D5 receptors in pain chronicity in male mice. J Neurosci 38:379–397 29167404 10.1523/JNEUROSCI.2110-17.2017 PMC5761615

[37] Huang S et al (2020) Dopamine inputs from the ventral tegmental area into the medial prefrontal cortex modulate neuropathic pain-associated behaviors in mice. Cell Rep 31:107812 32579938 10.1016/j.celrep.2020.107812

[38] Jääskeläinen SK et al (2001) Role of the dopaminergic system in chronic pain – a fluorodopa-PET study. Pain 90:257–260 11207397 10.1016/S0304-3959(00)00409-7

[39] Bouckoms AJ et al (1992) Monoamines in the brain cerebrospinal fluid of facial pain patients. Anesth Prog 39:201–208 7504420 PMC2148620

[40] Hagelberg N et al (2003) Striatal dopamine D1 and D2 receptors in burning mouth syndrome. Pain 101:149–154 12507709 10.1016/s0304-3959(02)00323-8

[41] Chudler EH, Dong WK (1995) The role of the basal ganglia in nociception and pain. Pain 60:3–38 7715939 10.1016/0304-3959(94)00172-B

[42] Wang X-Q, Mokhtari T, Zeng Y-X, Yue L-P, Hu L (2021) The distinct functions of dopaminergic receptors on pain modulation: a narrative review. Neural Plast 2021:6682275 33688340 10.1155/2021/6682275 PMC7920737

[43] Dieb W, Ouachikh O, Durif F, Hafidi A (2016) Nigrostriatal dopaminergic depletion produces orofacial static mechanical allodynia. Eur J Pain 20:196–205 25899074 10.1002/ejp.707

[44] Magnusso O, Fowler C, Kohler C, Ogren S (1986) Dopamine D2 receptors and dopamine metabolism relationship between biochemical and behavioural effects of substituted benzamide drugs. Neuropharmacology 25:187–197 2939362 10.1016/0028-3908(86)90040-7

[45] Boudier-Revéret M et al (2020) Association between chronic pain and alterations in the mesolimbic dopaminergic system. Brain Sci 10(10):701 33023226 10.3390/brainsci10100701 PMC7600461

[46] Ren W et al (2016) The indirect pathway of the nucleus accumbens shell amplifies neuropathic pain. Nat Neurosci 19:220–222 26691834 10.1038/nn.4199 PMC4889808

[47] Ren W et al (2021) Adaptive alterations in the meso-accumbal network after peripheral nerve injury. Pain 162:895–906 33021562 10.1097/j.pain.0000000000002092 PMC9272541

[48] Ziółkowska B (2021) The role of mesostriatal dopamine system and corticostriatal glutamatergic transmission in chronic pain. Brain Sci 11:1311 34679376 10.3390/brainsci11101311 PMC8533867

